# A case report on a patient suffering from recurrent vomiting episodes, whose condition improved markedly during pregnancy and breast feeding

**DOI:** 10.1186/1471-230X-6-28

**Published:** 2006-10-21

**Authors:** Bodil Ohlsson

**Affiliations:** 1 From the Department of Clinical Sciences, Division of Gastroenterology, Entrance 35, 205 02 Malmö, Lund University, Sweden

## Abstract

**Background:**

The normal physiology of the gastrointestinal tract has been only cursorily examined. Consequently, the pathophysiology of disturbances of the gastrointestinal functions is poorly known. Recurrent vomiting is one of many functional conditions for which it is difficult to find an explanation and to treat. In the following a case is described of a patient presenting with recurrent vomiting episodes, whose condition improved spontaneously during pregnancy and breast feeding.

**Case presentation:**

A woman with recurrent vomiting episodes over several years was examined by esophagogastroduodenoscopy. This showed a non-peristaltic ventricle. Treatment with the procinetic drug cisapride (Prepulsid^®^) improved the peristalsis and reduced the symptoms. During pregnancy and breast feeding, she was free of symptoms, in spite of having discontinued her medication with cisapride (Prepulsid^®^).

**Conclusion:**

The fact that the patient improved during pregnancy and breast feeding, would seem to indicate the involvement of factors in the physiology of pregnancy and breast feeding that are of importance for gastric motility. This deserves further investigation.

## Background

The normal motor function of the gut is controlled by the sympathetic and parasympathetic nervous system in addition to the enteric nervous system, interstitial cells of Cajal (ICCs) and smooth muscle cells. Severe gastrointestinal dysmotility, including gastroparesis, can develop as a result of the abnormal function of any of these systems including their associated neurotransmitters [1]. The pathogenesis may be immune-mediated, as in post-infectious gastroparesis [2], or may be due to defects in the ICCs, autonomic nervous system and smooth muscle, as in diabetic gastroparesis [3]. One third of all cases of gastroparesis are still regarded as idiopathic [4]. Several hormones and neurotransmitters have been suggested to be involved in this condition [5]. In clinical practice, I have noticed that some patients with severe gastrointestinal motility problems have improved during pregnancy and breast feeding.

In the following, I describe a woman with recurrent vomiting and reduced gastric motility, who was completely free of problems during pregnancy and breast feeding. A hypothesis could be raised on this finding, namely, that peptides and/or neurotransmitters appearing in altered concentrations during these conditions are involved in the physiology and pathophysiology of the gastrointestinal tract.

## Case presentation

A previously healthy woman, born in 1975, with no heredity for abdominal diseases, started to have attacks of vomiting during her teenage period. Eating late in the evening, caused her to wake up a few hours later with violent attacks of vomiting. To avoid vomiting, she had to avoid these situations and had to stay awake many hours after the last meal of the day. After some years she sought medical help for this problem. An esophagogastroduodenoscopy revealed an open cardia and a strikingly atonic, non-peristaltic ventricle. This gave rise to the suspicion of a defective emptying of the ventricle as a cause of the vomiting. The results of a histological examination of mucosal biopsies from the ventricle and duodenum were normal. Physical examination, including X-ray examination of heart and lungs, showed no abnormality. Routine blood samples were all within the normal range. She was not using any drugs. Thus, no etiology of the impaired motility could be identified.

The patient was treated with the procinetic drug cisapride (Prepulsid^®^). This drug improved her symptoms with reduced vomiting, and she was able to eat in the evening. Another esophagogastroduodenoscopy, done in the course of the treatment with cisapride, showed an improved, normalized peristalsis in the ventricle. After a few years she became pregnant. She had stopped the medication with cisapride when planning the pregnancy. A light nausea was present during the first three months of pregnancy, but there was no vomiting. Thus, in spite of discontinuing the drug treatment during this period, she had no vomiting episodes. This situation continued during breast feeding, which lasted about 7 months. However, three to four weeks after the breast feeding was terminated, her attacks of vomiting resumed, and cisapride (Prepulsid^®^) had to be reintroduced. When she was expecting her second child, the same sequence of events recurred. During pregnancy and breast feeding, which this time lasted for three months, there was no need of any procinetic drug. She had no nausea at all during this pregnancy. Just as after the first breast feeding, the vomiting resumed three to four weeks after the breast feeding was ceased. She did not make any changes in her dietary habits during or after her pregnancies and breast feedings.

The activity of the endocrine system of the gut is enhanced during pregnancy and lactation. Functionally, the effect of this enhanced activity is to contend with conditions of greater nutritional need by stepping up the digestive capacity and rendering the metabolism anabolic. Several of the peptides involved have been suggested to play a role in the etiology of idiopathic gastroparesis [5]. As the present patient was remarkably improved during pregnancy and breast feeding, the appropriate questions may be, which changes during these conditions also restitute impaired gastrointestinal motility, and which factors are expressed in insufficient or increased concentrations under normal conditions, leading to dysmotility.

The patient in question responded promptly to cisapride, an agent that increases acetylcholine release by acting on presynaptic serotonin (5-HT_4_) receptors [6]. This suggests that serotonin and/or acetylcholine are important in this dysfunction. However, it is not known whether either of these neurotransmitters, both of which are of importance for the stimulation of gastric motility [6,7], are expressed in higher levels during pregnancy or breast feeding. The ability to respond to therapy and pregnancy and breast feeding argues against irreversible structural defects.

Several hormones of importance for gastrointestinal motility are found at higher plasma concentrations during pregnancy. One example is motilin, an important peptide in digestive motility whose receptor is the site of action for erythromycin in the treatment of gastroparesis [7]. Motilin is elevated both during pregnancy and immediately postpartum [8], whereas plasma concentrations during breast feeding have to date not been examined. The motilin-related peptide ghrelin, which originates primarily in the stomach [9], has recently also been shown to enhance gastric emptying in idiopathic gastroparesis [10]. However, this peptide is expressed at lower concentrations during pregnancy than during normal conditions [11].

Other examples of hormones appearing in increased concentrations in the plasma during pregnancy are sex hormones, which are known to delay gastrointestinal transit time [12]. Furthermore, cholecystokinin (CCK) is elevated during pregnancy and gastrin during breast feeding [13,14]. These changes should rather impair gastric motility, as these hormones inhibit the gastric emptying during physiological conditions [15].

Oxytocin, a hormone with well-known stimulatory effects on myoepithelial cells and uterine smooth muscles, is elevated during pregnancy and breast feeding [16,17]. Recently, oxytocin has been shown to be present throughout the human gastrointestinal tract [18]. Furthermore, it is released postprandially in healthy subjects [19]. When the oxytocin receptor antagonist atosiban was administered in conjunction with a meal, the gastric emptying rate was prolonged [20]. Taken together, these findings indicate that oxytocin may play a physiological role also in the regulation of gastric motility.

Nitric oxide (NO) is one neurotransmitter assumed to be of interest in the etiology of diabetic gastroparesis [21]. However, NO production has been shown to be unaffected during pregnancy [22], suggesting that this is of minor interest in the case under consideration. One may further speculate about the altered profile of prostaglandins during pregnancy, but this has been only rudimentarily examined [23].

## Conclusion

Altogether, the hormonal and paracrine/autocrine changes during pregnancy and breast feeding are complex, and hormones with both stimulatory and inhibitory effects on gastric emptying and motility are released in higher amounts during these conditions. By studying physiological processes during pregnancy and breast feeding, we may learn more about the physiology and pathophysiology of the gastrointestinal functions. Serotonin, motilin and oxytocin may be of great interest, and development of new agonists to these peptides calls for further research.

## Competing interests

The author(s) declare that they have no competing interests.

## Pre-publication history

The pre-publication history for this paper can be accessed here:


